# The Bleeding Edge: Managing Coagulation and Bleeding Risk in Patients with Cirrhosis Undergoing Interventional Procedures

**DOI:** 10.3390/diagnostics14222602

**Published:** 2024-11-20

**Authors:** Rareș Crăciun, Cristiana Grapă, Tudor Mocan, Cristian Tefas, Iuliana Nenu, Alina Buliarcă, Horia Ștefănescu, Andrada Nemes, Bogdan Procopeț, Zeno Spârchez

**Affiliations:** 1 Department of Internal Medicine, “Iuliu Hațieganu” University of Medicine and Pharmacy, 400012 Cluj-Napoca, Romania; craciun.rares.calin@elearn.umfcluj.ro (R.C.); cristianagrapa@yahoo.com (C.G.); alinasauciuc@yahoo.com (A.B.); bogdan.procopet@umfcluj.ro (B.P.); zsparchez@gmail.com (Z.S.); 2 Gastoenterology Clinic, “Prof. Dr. O. Fodor” Regional Institute of Gastroenterology and Hepatology, 400162 Cluj-Napoca, Romania; horia.stefanescu@irgh.ro; 3 UBBmed Department, Babeș-Bolyai University, 400084 Cluj-Napoca, Romania; 4 Department of Physiology, “Iuliu Hațieganu” University of Medicine and Pharmacy, 400012 Cluj-Napoca, Romania; iuliana.nenu@gmail.com; 5 2nd Department of Anesthesia and Intensive Care, “Iuliu Hațieganu” University of Medicine and Pharmacy, 400012 Cluj-Napoca, Romania; andrada.nemes@ymail.com; 6 Intensive Care Unit, Cluj-Napoca Municipal Hospital, 400139 Cluj-Napoca, Romania

**Keywords:** cirrhosis, coagulation, bleeding, transfusion, viscoelastic tests, interventional procedures, endoscopy, paracentesis

## Abstract

This review addresses the peri-procedural bleeding risks in patients with cirrhosis, emphasizing the need for careful coagulation assessment and targeted correction strategies. Liver disease presents a unique hemostatic challenge, where traditional coagulation tests may not accurately predict bleeding risk, complicating the management of procedures like paracentesis, endoscopic therapy, and various interventional procedures. As such, this paper aims to provide a comprehensive analysis of current data, guidelines, and practices for managing coagulation in cirrhotic patients, with a focus on minimizing bleeding risk while avoiding unnecessary correction with blood products. The objectives of this review are threefold: first, to outline the existing evidence on bleeding risks associated with common invasive procedures in cirrhotic patients; second, to evaluate the efficacy and limitations of standard and advanced coagulation tests in predicting procedural bleeding; and third, to examine the role of blood product transfusions and other hemostatic interventions, considering potential risks and benefits in this delicate population. In doing so, this review highlights patient-specific and procedure-specific factors that influence bleeding risk and informs best practices to optimize patient outcomes. This review progresses through key procedures often performed in cirrhotic patients. The discussion begins with paracentesis, a low-risk procedure, followed by endoscopic therapy for varices, and concludes with high-risk interventions requiring advanced hemostatic considerations. Each chapter addresses procedural techniques, bleeding risk assessment, and evidence-based correction approaches. This comprehensive structure aims to guide clinicians in making informed, evidence-backed decisions in managing coagulation in cirrhosis, ultimately reducing procedural complications and improving care quality for this high-risk population.

## 1. Background and Rationale

Patients with cirrhosis have long been considered to have a high risk of bleeding. This paradigm had a twofold mechanistic explanation. On the one hand, it was observed that these patients have persistently abnormal standard coagulation tests (prolonged international normalized ratio—INR; low platelet count; low fibrinogen levels). On the other hand, they tend to have more frequent bleeding events (gastrointestinal bleeding, portal hypertension-related or not, post-procedural bleeding events, frequent bruising). However, evidence emerging in the past two decades has debunked both epistemological pathways.

First, the abnormal standard coagulation profile frequently encountered in patients with advanced liver disease often shows only one facet of the hemostatic balance, as none of these variables can provide a global assessment of hemostasis. Thus, the liver is an organ with a pivotal role in regulating coagulation, producing not only procoagulant factors (their deficit being reflected in an increased INR) but also anticoagulant molecules (protein C, protein S, and antithrombin) [1]. Moreover, a low platelet count is not only a direct consequence of splenic sequestration due to portal hypertension but is also partially determined by low thrombomodulin production and an increased resistance to its effects [2,3]. Not least, thrombocytopenia is rebalanced by an increased level of activation due to an increase in the von Willebrand factor, a decrease in ADAMTS-13, and platelet activation due to portal hypertension-induced endotoxemia [4,5].

Secondly, the higher rate of bleeding events in patients with cirrhosis is explained in a large part by portal hypertension-related bleeding events, which are a function of the increased intravascular pressure exceeding the elastic properties of the vessel, in accordance with Laplace’s Law [6], a higher rate of non-variceal upper gastrointestinal bleeding due to concurrent risk factors [7], significant frailty leading to trauma and bruising [8], and a greater need for interventional procedures throughout the disease course (endoscopic therapy, liver biopsies, transjugular intrahepatic porto-systemic shunt placement, paracentesis, or thoracocentesis).

In this light, given a better understanding of the concept of rebalanced hemostasis among the community of hepatologists, there is still an unmet need to harmonize the scientific output and basic evidence with factual, actionable recommendations for clinical practice. To this point, there is a lack of validated tools to assess the bleeding risk, especially in relation to interventional procedures, as global hemostatic tests such as thromboelastography or rotational thromboelastometry have yet to become a widely available standard of care and still require extensive validation [9,10].

Recently, two of the major gastroenterology and hepatology associations, namely the American Gastroenterology Association (AGA) [11] and the European Association for the Study of the Liver (EASL) [12], have published clinical practice guidelines on the topic of bleeding risk in cirrhosis. While these documents are certainly comprehensive, data-ridden, and extremely valuable, our team felt that a clinically oriented approach for the most frequent invasive procedures is still lacking. Consequently, we constructed a procedural-based review of the available data, in an attempt to provide a fact-based rationale for assessing and mitigating bleeding risk for the day-to-day use of practicing gastroenterologists, hepatologists, and interventionists, focusing on the most common procedures: paracentesis, liver biopsy, percutaneous ablation, endoscopic therapy of esophageal varices, colonoscopy, and endoscopic retrograde cholangiopancreatography (ERCP).

## 2. Bleeding Risk in Paracentesis—The Most Frequent Clinical Dilemma

According to the EASL guidelines, thrombocytopenia, defined using various thresholds to denote severity, has not consistently demonstrated an increased risk of bleeding following various minimally invasive procedures, including paracentesis, thoracentesis, dental extraction, central venous cannulation, hepatic venous pressure gradient measurement, esophageal variceal band ligation, endoscopic polypectomy, and transjugular or laparoscopic liver biopsy [12]. However, low fibrinogen levels have been linked to an elevated risk of bleeding after paracentesis in patients with acute-on-chronic liver failure [12]. Notably, a fibrinogen level below 60 mg/dL emerged as the strongest independent predictor of new-onset major bleeding episodes in a cohort of 1493 critically ill cirrhotic patients. However, a causative relationship was not definitively established, with most bleeding events attributable to portal hypertension [13].

In decompensated cirrhosis, acute kidney injury (AKI) is linked to increased bleeding risk during procedures like paracentesis. More than 30 years ago, McVay et al. found that patients with elevated serum creatinine levels, primarily due to AKI, had a significantly higher risk of bleeding during paracentesis or thoracentesis [14]. Similarly, in a recent case–control study, AKI but not sepsis prior to paracentesis was the most significant predictor of bleeding, independent of factors like INR, platelet count, or MELD score. Another study showed that all patients who bled during procedures had concurrent AKI and/or bacterial infections, while baseline thrombocytopenia or INR prolongation did not correlate with bleeding risk [15].

However, one study demonstrates the activation of coagulation and fibrinolysis within the peritoneal cavity of cirrhotic patients with ascites [16]. While tissue factor-exposing extracellular vesicles in ascitic fluid remain localized, plasminogen activation to plasmin occurs within the ascitic fluid, potentially re-entering circulation and triggering systemic hyperfibrinolysis. This process may contribute to the development of acute-on-chronic liver failure (ACLF), commonly observed in decompensated cirrhosis, as well as nonportal hypertensive upper gastrointestinal bleeding events, which are associated with high mortality rates.

The risk of bleeding post-minimally invasive procedures in cirrhotic patients encompasses factors extending beyond alterations in hemostatic parameters, emphasizing technical considerations and procedure-specific factors amenable to intra-procedural mitigation through reactive, noncoagulation-related measures (e.g., dental extractions, endoscopic polypectomy). Moreover, the bleeding risk associated with a given procedure may vary, contingent upon distinct clinical contexts, such as stable disease versus concurrent acute events like sepsis or acute kidney injury. For instance, in patients with stable cirrhosis and abnormal coagulation profiles (mean platelet count 50.4 × 10^9^/L, mean INR 1.7), no post-paracentesis bleeding incidents were documented, while a 3% post-paracentesis bleeding rate was observed in patients with ACLF undergoing the same procedure, despite a similar standard coagulation profile [17]. In addition, when patients with acute-on-chronic liver failure were propensity score-matched to those with decompensated cirrhosis based on liver disease severity, platelet count and INR did not significantly differ between individuals experiencing post-procedural bleeding and those who did not [18]. Hypofibrinogenemia emerged as the sole independent predictor of post-paracentesis bleeding [18].

In conclusion, the risk of bleeding following minimally invasive procedures in cirrhotic patients is influenced by a range of factors beyond traditional hemostatic parameters. Thrombocytopenia alone does not consistently predict bleeding; however, low fibrinogen levels and AKI have emerged as significant predictors, particularly in the context of ACLF. These findings underscore the necessity of considering specific clinical contexts and underlying conditions. Therefore, a comprehensive approach that integrates technical considerations and patient-specific factors is essential for minimizing bleeding risks in this patient population.

## 3. Liver Biopsy and Percutaneous Tumor Ablation

Liver biopsy continues to be a common necessity for patients with chronic liver disease. The occurrence of clinically evident bleeding complications ranges from 1 to 6 per 1000 biopsies [19]. Moreover, upon conducting systematic ultrasound assessments post-biopsy, indications of bleeding, such as liver hematoma or peritoneal fluid, are detected in 3–23% of patients [20,21]. There persists a significant gap in identifying individuals prone to bleeding associated with liver biopsy. Studies have shown that the predictive value of SCTs is poor in predicting post-procedural bleeding in cirrhotic patients [22].

A recent study on 302 patients undergoing liver biopsy revealed that employing previously established thresholds such as a platelet count below 50 × 10^9^ or an INR > 1.8 similarly failed to differentiate between patients who experienced procedure-related bleeding and those who did not [23]. In a multicenter prospective study examining 380 cirrhotic patients, both with and without abnormal coagulation parameters (INR of ≥1.5 and/or a platelet count of ≤50 × 10^9^/L) undergoing percutaneous liver biopsy, three patients from the abnormal coagulation subgroup encountered clinically significant bleeding, albeit without reaching statistical significance (*p* = 0.061). Notably, all three patients with such bleeding had Child–Pugh C cirrhosis, elevated INR, low platelet count, and additional contributing factors such as sepsis or acute kidney impairment (AKI) [24]. Additionally, a retrospective study on 1740 patients undergoing percutaneous liver biopsy revealed that the adoption of less stringent pre-procedural coagulation parameter criteria, with an INR threshold of ≤2.0 and platelet count of ≥25,000 µL, did not lead to a rise in departmental hemorrhagic complication rates. However, it notably reduced the requirement for pre-procedural fresh frozen plasma (FFP) and platelet transfusions [25].

Given that severe thrombocytopenia often mirrors advanced liver disease, negating the necessity for a histological confirmation of cirrhosis presence, and due to perceived bleeding risks, percutaneous liver biopsy in clinical practice is typically reserved for patients without portal hypertension and with a platelet count exceeding 50 × 10^9^/L. The HALT-C trial [19] encompassed the largest cohort of patients with advanced liver disease undergoing percutaneous liver biopsy. Although bleeding complications were rare (overall hemorrhagic rate—0.6%), the study underscored an elevated risk of post-procedural bleeding among patients with a platelet count of ≤60 × 10^9^/L (5.3%) compared to those with a platelet count exceeding 60 × 10^9^/L (0.4%). Recent investigations into thrombopoietin receptor agonists (TPO-RAs) have demonstrated their efficacy in diminishing the necessity for platelet transfusions among cirrhotic patients undergoing elective invasive procedures. It is conceivable that TPO-RAs offer a more potent hemostatic effect compared to platelet transfusions [26,27]. The recommended treatment duration preceding biopsy is typically 9–14 days. Although not consistently observed across all studies, the use of TPO-RAs has been linked to an elevated likelihood of thrombotic events, including portal vein thrombosis [25]. Therefore, their administration demands prudence, especially in patients deemed to be at a heightened risk of thrombosis.

Taking into account all recent data, the EASL guidelines do not recommend routine assessments of hemostatic parameters in cirrhotic patients undergoing liver biopsy. Recommendations from other guidelines regarding pre-procedural assessments of patients who present with a liver biopsy indication are summarized in Table 1.

Regarding liver ablation, in clinical practice, percutaneous ablation (radiofrequency ablation—RFA; or microwave ablation—MWA) for HCC is seldom carried out in individuals with platelet counts below 50 × 10^9^/L. Instead, such cases typically involve platelet transfusions and vigilant platelet count monitoring prior to the procedure. Consequently, the incidence of post-procedural bleeding for HCC remains below 1% [32,33]. Only one study by Park et al. [32] identified a link between platelet counts below 50 × 10^9^/L and an elevated risk of post-procedural bleeding (OR = 8.79). However, this finding may be influenced by the prophylactic platelet transfusions administered to patients with platelet counts below 50 × 10^9^/L, potentially introducing bias. Research conducted by Goto et al. [34] revealed a hemorrhagic complication rate of 1.5% across 4133 radiofrequency ablations. Although patients with severe thrombocytopenia (platelet counts < 50 × 10^9^/L) were not included in the study, thrombocytopenia was still identified as a significant risk factor for hemoperitoneum. Despite liver ablation being considered a procedure with a low risk of bleeding, Cardiovascular and Interventional Radiology Society of Europe (CIRSE) guidelines still recommend that all patients should have a platelet count > 50.000 and an INR < 1.5 [35], with patients with a lower platelet count often receiving platelet transfusions. The pre-procedural practice of platelet transfusion lacks standardization, as neither a definitive threshold prompting transfusion nor an optimal target platelet count has been determined. Additional large-scale studies are needed to revise pre-procedural coagulation guidelines in this particular clinical scenario.

Mounting evidence indicates that viscoelastic assays offer a valuable means of evaluating the hemostatic status in patients with liver disease, potentially resulting in a decreased utilization of blood products. In a small, randomized trial [36], it was demonstrated that among patients with cirrhosis requiring invasive procedures and presenting with an INR > 1.8 and/or a platelet count < 50 × 10^9^/L, a thromboelastogram-guided transfusion strategy significantly reduced blood product utilization (17% compared to 100% in the standard care group), without an increase in bleeding complications. Similar findings have been observed in other open-label randomized studies [36,37]. Consequently, viscoelastic testing has emerged as a successful approach for curbing unnecessary prophylactic transfusions in liver disease patients, surpassing the traditional reliance on the INR and platelet count. It is important to acknowledge, however, that these assays have yet to demonstrate consistent reliability in predicting bleeding events among patients with liver disease.

In conclusion, percutaneous liver biopsy/ablation, being invasive procedures, carry potential clinical complications. These factors impact both payer and healthcare costs, necessitating precise estimates of complications to guide clinical guidelines effectively. Research indicates that in individuals with chronic liver disease, including those at advanced stages, relying solely on platelet count or INR levels as a predictor of increased bleeding risk is insufficient. Instead, SCTs should be assessed in conjunction with other risk factors such as sepsis and acute kidney injury to provide a more precise evaluation of the patient’s bleeding risk. Furthermore, in cirrhotic patients, both the underlying cause and severity of the disease can impact the delicate balance of hemostasis. Additionally, we must take into account the futility of interventional procedures in advanced liver disease patients; given their fragile balance and potential coexisting conditions, the aforementioned procedures are rendered obsolete.

## 4. Acute Variceal Bleeding and Endoscopic Therapy of Varices

Acute variceal bleeding (AVB) is responsible for 70% of clinically significant upper gastrointestinal bleeding episodes in patients with portal hypertension. AVB represents the second most common decompensating event following ascites and stands as one of the most severe and immediately life-threatening complications in a cirrhotic patient [38]. There is strong evidence indicating that AVB is not associated with hemostatic dysfunction, and its occurrence is directly correlated with the severity of portal hypertension and local vascular irregularities. In this light, the degree of portal hypertension expressed by the hepatic venous pressure gradient (HVPG) is strongly correlated with the risk of developing varices, AVB, and failure to successfully control an episode of AVB using first-line therapy [6,39,40,41,42].

The cornerstone of AVB treatment is endoscopic therapy in combination with pharmacological therapy, which aims to decrease portal pressure, improve endoscopic therapy outcomes, and prevent further complications [6,38]. In most cases, endoscopic therapy consists of variceal band ligation (VBL), which, according to the 2022 European Association for the Study of the Liver (EASL) guidelines on the management of bleeding and thrombosis, is considered a high-risk invasive procedure [12]. The bleeding risk following VBL ranges between 2.7% and 7.3%. However, most of the bleeding episodes occur late after the procedure, either due to the inadequate control of the decompensating event and underlying portal hypertension or because of the mechanical injury produced by the natural dislodgement of the endoscopic bands. Neither standard coagulation tests (PT, INR, platelet count, fibrinogen levels) nor viscoelastic tests are reliable predictors of peri-procedural bleeding [43,44,45,46], with only one relatively small-scale retrospective study reporting an association between low fibrinogen levels and rebleeding after VBL [47].

Given that coagulation test abnormalities are frequently encountered in patients with AVB, and neither test is associated with bleeding control or rebleeding, it is sensible to assume that correcting these abnormalities does not improve peri-procedural outcomes. Moreover, there is evidence that not even anticoagulant therapy increases the risk of AVB, and it does not influence rebleeding rates in these patients, further reinforcing the non-hemostatic character of this clinical event [48,49]. Consequently, using pro-hemostatics to manage variceal bleeding does not stand on solid ground. However, despite multiple consensus recommendations and expert opinions [12,50,51,52,53], patients with AVB are still the recipients of unwarranted blood products, especially fresh frozen plasma (FFP), platelet units, and excessive red blood cell transfusions. Concerningly, the detrimental effects of unjustifiably using blood products expand well beyond futility, economics, and societal costs. According to a multicentric study published in 2021, patients with AVB receiving FFP had significantly worse outcomes, with increased 42-day mortality (odds ratio—OR—9.41, 95% confidence interval—CI—3.71–23.90), 5-day failure to control bleeding (OR 3.87, 95% CI 1.28–11.70), and length of hospitalization beyond 7 days (OR 1.88, 95% CI 1.03–3.42) [54]. In addition, data from the MIMIC-IV database published in 2024 have shown that in patients with decompensated cirrhosis admitted to an intensive care unit, FFP is associated with higher odds of liver, kidney, coagulation, respiratory, and circulatory failure, without any improvement in survival [55]. A similar pattern occurs regarding platelet transfusion. A study that included 913 patients in a prospectively maintained database has shown that patients receiving platelets had higher 5-day and 42-day rebleeding rates (14.6% vs. 4.5%; *p* = 0.039, and 32.6% vs. 15.7%; *p* = 0.014, respectively), without any significant impact on mortality, while also reporting that thrombocytopenia did not influence rebleeding or mortality [56].

A similar principle of futility in correcting SCT abnormalities (INR > 1.5 or platelet count < 50.000/mm^3^) was demonstrated in patients undergoing elective VBL. A large-scale study that included over 1000 VBL procedures, primarily performed in an outpatient setting, showed that the incidence of post-VBL bleeding was relatively low (2.2%—33 out of 1472 procedures) and was not influenced by abnormal SCTs or blood product transfusion. Instead, the most reliable predictor for post-procedural bleeding was liver disease staging (Child–Pugh class and MELD scores), reinforcing that bleeding is rather influenced by the degree of portal hypertension instead of SCT abnormalities [57].

In conclusion, there is strong evidence that an increased INR and thrombocytopenia are poor predictors for bleeding risk and AVB control, and thus, correcting these abnormalities using blood products is not only ineffective but can even be detrimental. Moreover, deciding whether or not to perform the endoscopic therapy of varices should not be influenced by standard coagulation tests.

## 5. Colonoscopy and Polyp Removal

Colonoscopy is a widely employed method for diagnosing and treating colonic diseases, particularly colorectal neoplasia. Despite its overall safety and effectiveness, it carries certain risks of side effects and complications, including bleeding, perforation, infection, and cardiovascular events. While bleeding after diagnostic colonoscopy is rare, it can occur and is often associated with biopsy procedures. This complication may arise due to the direct sampling of blood vessel structures, particularly in individuals with impaired blood coagulation, such as those with liver cirrhosis (LC) [58,59]. Additionally, mechanical friction caused by the endoscope can contribute to bleeding, although this is uncommon. According to Kavic et al., the incidence of bleeding during diagnostic colonoscopies is a mere 0.03% [58,59].

Polyp removal during colonoscopy is crucial for reducing the incidence and mortality rates of colorectal cancer. However, this procedure also carries risks, including bleeding, perforation, and post-polypectomy syndrome. Bleeding is the most common complication, with reported frequencies ranging from 0.3% to 6.1% [59,60,61]. The occurrence of bleeding can be immediate or delayed, depending on various factors, including the morphology of the polyp. Pedunculated polyps typically have a large central vessel traversing through the stalk. Inadequate electrocoagulation during the cutting of the stalk with a snare can lead to significant pulsatile bleeding. Conversely, with sessile polyps, bleeding may occur from the internal edge of the section or from exposed vessels in the submucosal layer [59,62,63].

Applying appropriate prophylactic and therapeutic hemostatic techniques, such as thermal ablation, clips, or injected/topical hemostatic agents, is crucial. This is particularly true in patients with LC, where factors such as portal hypertension-induced hypersplenism and reduced hepatic synthesis of thrombopoietin and coagulation factors can lead to low platelet counts and impaired coagulation [43]. Patients with LC have a higher risk of bleeding compared to the general population, as evidenced by several studies. For example, a study including 1267 subjects found that early post-polypectomy bleeding (PPB) occurred significantly more often in the Child–Pugh B or C group (17.5%) than in the Child–Pugh A (6.3%) or non-LC (4.6%) groups [64]. Another retrospective study of 307 patients with LC reported an overall incidence of immediate PPB of 7.5% [65]. Furthermore, the incidence of delayed PPB was significantly higher in patients with LC compared to those without (13.8% vs. 4.2%) in another study [66]. Interestingly, a younger age, a higher MELD-Na score, a platelet count < 50,000/µL, and the presence of ascites or varices are also compounding risk factors for PPB [43,64,65,67].

Regarding advanced techniques, such as endoscopic mucosal resection or endoscopic submucosal dissection, in a systematic review and meta-analysis including ten studies with a total of 3244 patients with LC, the pooled rates of immediate and delayed bleeding were 9.5% and 6.6%, respectively [68]. A longer procedure duration, a lesion diameter >15 mm, and increased age seem to incur a higher bleeding risk [69]. Although these advanced techniques can result in frequent bleeding, especially in patients with LC, they offer the advantage of managing it meticulously through the application of a coagulation grasper with a gentle coagulation effect to address any visible vessels in the resection area [70].

## 6. Endoscopic Retrograde Cholangiopancreatography

Endoscopic retrograde cholangiopancreatography (ERCP) is a hybrid endoscopic and fluoroscopic technique used in the treatment of various hepatobiliary and pancreatic diseases such as stones or strictures. While it is a pivotal procedure, it is not without risks, with an estimated 6.85% of patients experiencing complications such as pancreatitis, bleeding, perforation, and/or infection, with up to a quarter of these being severe [71]. Encountered in 1.34% of patients, bleeding can ensue either during or after the procedure and can be caused by mechanical trauma during scope insertion, sphincterotomy, sphincteroplasty, or biopsy. Risk factors for bleeding include end-stage renal disease, anticoagulant intake, or cirrhosis [72,73,74].

In patients with cirrhosis, portal hypertension contributes to the formation of fragile blood vessels in the mucosa and submucosa of the gastrointestinal tract, increasing the likelihood of bleeding during invasive procedures. These factors, combined with mucosal fragility and susceptibility to trauma, highlight the heightened bleeding risk in cirrhotic patients undergoing ERCP. Thrombocytopenia, congestive duodenopathy, and coagulopathies are independent risk factors for bleeding [75].

In a comprehensive multicenter study encompassing 3228 cirrhotic patients, the incidence of ERCP-related bleeding was notably higher among cirrhotic individuals compared to non-cirrhotic counterparts (2.1% vs. 1.2%). Upon multivariable analysis, factors independently associated with an elevated risk of bleeding included decompensated cirrhosis (adjusted odds ratio [aOR] 2.7), compensated cirrhosis (aOR 2.2), and performing biliary sphincterotomy (aOR 1.6). Conversely, performing ERCPs in large- (aOR 0.56) and medium-sized (aOR 0.7) hospitals was associated with a reduced risk of post-procedure bleeding [76]. A higher risk of bleeding in decompensated cirrhosis, especially in Child–Pugh C class patients, was also demonstrated in several other studies [72,73,77]. Additionally, patients with a MELD-Na score >11.5, an APRI level of >1.49, or a New Wilson Index score >5 have higher rates of complications and mortalities [75,78]. However, it is to be noted that other studies reported no significant association found between post-sphincterotomy bleeding and the severity of liver cirrhosis [78,79,80,81].

A platelet count < 50,000/mm^3^, which can be encountered in patients with liver cirrhosis and hypersplenism, can further increase the risk of bleeding (OR 35.3) [74]. Despite the widespread use of INR as a standard test to evaluate bleeding risk before endoscopic procedures, a notable correlation between elevated INR levels and the occurrence of post-ERCP bleeding was not found [75]. This could be attributed to the elevated von Willebrand factor levels in cirrhosis, which facilitate platelet adhesion and activation, as well as sustained thrombin production [43].

Patients with liver cirrhosis and portal hypertension are also at risk of developing varices of the upper gastrointestinal tract, which are prone to bleeding. However, in patients undergoing ERCP, the risk of esophageal or gastric variceal bleeding seems to be negligible [82,83].

## 7. Tailored Approaches to Bleeding Challenges in Cirrhosis Patients

Historically, bleeding complications among cirrhosis patients were frequently attributed to alterations in their hemostatic profile, such as a prolonged prothrombin time or low platelet count, which were formerly construed as indicative of hypocoagulability [50]. Nowadays, the notion of rebalanced hemostasis in individuals with liver disease has prompted adjustments in the approach to prophylactic hemostatic intervention to some degree [4,84,85].

While various reports detail expert recommendations for prophylactic pro-hemostatic therapy in cirrhosis patients, insufficient attention has been paid to managing active bleeding in this population. This gap persists despite the recognition that bleeding in cirrhosis may not stem directly from hemostatic dysfunction and that hemostatic equilibrium is typically upheld [86]. Consequently, devising strategies to address active bleeding in these patients proves intricate and may not invariably necessitate pro-hemostatic interventions.

### 7.1. Prevention of Bleeding

Before any procedure, the initial step in preventing bleeding complications is to assess the risk of bleeding. This process should commence as soon as the procedure is scheduled and be revisited promptly before the procedure. Clinicians should be aware of the patient’s medical history, have a clear rationale for indicating a certain procedure, and assess the use of laboratory tests in this particular category of patients. According to recent guidelines [12], procedural bleeding risk in patients with cirrhosis has been categorized as low-risk (<1.5%), which includes paracentesis, thoracocentesis, trans-esophageal echocardiography, percutaneous liver biopsy, transjugular liver biopsy, HVPG measurement, and percutaneous ablation of liver cancer, and high risk includes the following (>1.5%): ERCP, endoscopic polypectomy, endoscopic esophageal varices ligation, and dental extraction. As a precaution against bleeding risks alone, prophylaxis for low-risk procedures is generally not advised [22]. Nonetheless, it may be considered in specific instances under the guidance of the attending physician. Furthermore, the operator’s expertise and the procedure’s technical complexity are pivotal considerations that should not be overlooked.

Standard coagulation assessments prior to invasive procedures typically involve a blood cell count, prothrombin time, and activated partial thromboplastin time (APTT) [87]. However, the international normalized ratio (INR) and APTT lack predictive value for post-procedural bleeding in cirrhosis patients undergoing invasive procedures. Regarding platelet count, while studies fail to establish a reliably safe threshold associated with bleeding risk, a platelet count exceeding 50 × 10^9^/L is commonly deemed acceptable for conducting invasive procedures [22,88].

When planning the procedure, another critical measure involves identifying potentially modifiable risk factors for bleeding, like using anticoagulants or antiplatelet agents and assessing indicators of renal impairment or sepsis [89]. Studies suggest that decompensating events, such as acute kidney injury, infection, and disease progression, coincide with hypocoagulable alterations that exacerbate bleeding tendencies [15,89].

There exists considerable diversity in how major bleeding is defined across source publications, with varying approaches to correcting hemostatic markers and an inconsistent utilization of imaging guidance. The EASL guidelines advise against assessing hemostatic markers before low-risk bleeding procedures but propose that these markers could provide a valuable baseline before high-risk procedures. This baseline can aid in assessing the extent of liver disease severity and guide the management of subsequent peri-procedural bleeding. Table 2 provides a comparison between the two guidelines, EASL and AASLD (101), regarding pre-procedural bleeding risk assessment and the correction of coagulation parameters for patients with liver cirrhosis undergoing invasive procedures (low-risk or high-risk).

### 7.2. Treatment of Bleeding

Both the EASL and AASLD guidelines outline three primary causes of bleeding in cirrhosis patients: (1) bleeding complications related to portal hypertension, such as variceal bleeding, which appear to be independent of hemostatic failure, as supported by significant evidence; (2) bleeding due to procedures that lead to mechanical vessel injury; such events are also not primarily attributable to hemostatic failure, thus rendering prophylactic procoagulant therapy unlikely to prevent them; and (3) spontaneous or unprovoked bleeds, such as bruising, mucosal bleeding, and oozing from puncture sites, may be linked to hemostatic failure and are challenging to predict [12,51].

When bleeding occurs, clinicians often resort to pro-hemostatic treatments involving blood products, factor concentrates, or antifibrinolytics. However, given that many bleeding scenarios in cirrhosis patients are not related to hemostatic failure, there is debate on whether such treatment should be initiated initially [90]. It has been suggested that pro-hemostatic therapy may not constitute the initial therapeutic approach in many common bleeding scenarios in cirrhosis patients, as alternative strategies may offer better management depending on the specific circumstances [91]. Recommendations regarding different bleeding scenarios in cirrhotic patients according to the latest EASL guidelines are summarized in Table 3. In cirrhotic patients with variceal bleeding, the efficacy of vasoactive drugs (terlipressin/octreotide) and the ineffectiveness of procoagulant factors act as a testimony for the preferred restrictive approach applied in this category [92]. Recent studies have shown that the use of products such as fresh frozen plasma or tranexamic acid for variceal bleeding is associated with worse outcomes [93,94]. The use of pro-hemostatic measures in bleeding scenarios is justified by a few clinical scenarios, such as patients experiencing intractable bleeding or those undergoing transplant surgery with significant bleeding. In such cases, low-volume products like fibrinogen concentrate and prothrombin complex concentrate are preferable to fresh frozen plasma and platelet concentrate [93]. This preference arises from concerns that the latter options could elevate portal pressure and worsen bleeding events associated with portal hypertension. In summary, bleeding events related to portal hypertension should be addressed with pharmacological interventions to reduce portal pressure, along with local measures to control bleeding, and mechanical bleeding events may be managed with local measures such as applying pressure for dental extraction-related bleeding events or vessel ligation during surgery [12,51,92].

### 7.3. Assessing Coagulation

Cirrhosis creates a unique hemostatic environment characterized by a delicate equilibrium between procoagulant and anticoagulant forces. This balance, influenced by platelet dynamics, coagulation factors, and systemic inflammation, complicates the interpretation of standard coagulation tests and necessitates advanced diagnostic approaches [1]. Given that standard coagulation tests have limited applicability in assessing the hemostatic balance of cirrhotic patients, the most pressing knowledge gap is in finding a solid alternative. The subsequent brief discussion aims to dissect this issue, starting with each of the standard coagulation variables.

Platelet count in cirrhosis is reduced due to the following: (a) decreased production: impaired thrombopoietin synthesis by the liver results in reduced megakaryocyte stimulation; (b) increased clearance: hypersplenism due to portal hypertension accelerates platelet sequestration and destruction; and (c) immune activation: autoantibodies and immune complex-mediated platelet clearance further exacerbate thrombocytopenia. Paradoxically, despite low platelet counts, platelet function can exhibit compensatory hyperactivation. Elevated von Willebrand factor (vWF), due to reduced ADAMTS-13 activity, enhances platelet adhesion, often masking functional deficits. These shifts contribute to a state of unstable counterbalancing, where both hypo- and hypercoagulable tendencies coexist [4,5,95]. Moreover, the current methods for assessing platelet count and function are susceptible to multiple potential errors, ranging from preanalytical (delayed blood processing or inappropriate anticoagulant use can artificially alter platelet activity or citrate-induced dilution effects, particularly pronounced in thrombocytopenic patients, can lead to inaccurate results) to technical (impedance-based analyzers often misclassify small, hyperactive platelets seen in cirrhosis, leading to an underestimation of functional capacity) or clinical modifiers (medications like beta-blockers reduce platelet aggregation, while metabolic factors such as hyperbilirubinemia impair platelet energy metabolism and function) [1]. To counterbalance these caveats, multiple methods provide a more in-depth assessment of platelet function. Still, they are prone to various limitations that limit their availability and day-to-day use. One such method is aggregometry, which measures platelet aggregation in response to agonists like ADP or collagen. Aggregometry is highly sensitive to platelet function changes but has the limitations of being labor-intensive and prone to preanalytical variability. Other techniques are lumiaggregometry, which combines aggregation measurements with ATP release detection for assessing dense granule secretion; flow cytometry, which evaluates surface activation markers (such as P-selectin) and circulating platelet-monocyte aggregates; and microfluidic systems, which evaluate platelet function under physiological flow conditions, thus better simulating in vivo environments [96].

Regarding PT (or INR) and aPTT, these methods were designed to detect hematological disorders and to guide anticoagulant therapy. A singular unidirectional pathway alteration characterizes both scenarios, namely a deficit in procoagulant factors (either congenital, acquired, or blocked for therapeutic purposes). Hence, these metrics do not take into account any counterbalancing response and cannot assess any corresponding deficit in anticoagulant factors. Therefore, PT and aPTT are inadequate in assessing complex alterations in hemostasis, like the rebalanced hemostasis of patients with advanced liver disease, which have both deficits in coagulation factors (II, V, VII, IX, X, XI) which lead to a prolonged PT and aPTT, as well as deficits in anticoagulant factors (protein C, S, and antithrombin) which are not measured by standard coagulation tests [1]. This limitation can be overcome by thrombin generation tests, which are sensitive to natural anticoagulant factors. While conventional thrombin generation assays are susceptible to errors due to an inadequate assessment of the protein C pathway, more recent modified tests, which include the addition of thrombomodulin, are sensitive to all pro- and anticoagulant pathways and can provide a more accurate assessment of the rebalanced hemostasis [85]. Thus, by using such assays, multiple reports have shown that the hemostatic equilibrium remains preserved even in severe liver dysfunction, such as ACLF [97,98]. However, thrombin generation assays also have significant limitations due to the lack of standardization, preanalytical variability, and the inability to fully capture the complex balance between pro- and anticoagulant factors. Elevated factor VIII levels in cirrhosis may skew the results, and platelet contributions or cirrhotic plasma characteristics (e.g., high bilirubin) are often not reflected. The assays require specialized equipment, are costly, and may not correlate well with clinical outcomes. Artificial assay conditions, such as added tissue factors, further limit physiological relevance [99].

Patients with cirrhosis have significantly lower levels of fibrinogen, and evidence suggests that fibrin polymerization is also impaired, thus contributing to an increase in thrombin time [1]. On the other hand, despite lower levels and poor polymerization, there is evidence that once the clot is formed, its strength is significantly higher, thus generating a compensatory procoagulant response [100]. These dynamic alterations in fibrinogen function pose significant difficulties for routine assessment.

Viscoelastic tests (VETs), such as rotational thromboelastometry (ROTEM), thromboelastography (TEG), and more recently ClotPro, play a significant role in assessing coagulation in patients with cirrhosis. These tests provide real-time, comprehensive evaluations of the clotting process, including clot initiation, formation, strength, and dissolution, offering insights beyond standard coagulation tests. VETs are particularly useful in cirrhosis because they measure the functional balance of pro- and anticoagulant factors rather than isolated components. They allow for whole-blood analysis, capturing contributions from clotting factors, anticoagulants, platelets, and fibrinogen. Key metrics include clotting time (CT), which assesses initiation; maximum clot firmness (MCF), reflecting clot strength; and lysis indices, indicating fibrinolysis. While these tests do not give specific insights into each component of the hemostatic process, they compensate by providing a global overview. Viscoelastic tests can guide transfusion therapy, identifying deficiencies in fibrinogen or platelets and reducing unnecessary blood product use, especially before interventional procedures [10,101,102,103]. However, these tests have limitations. They require specialized equipment and expertise, and cirrhotic alterations, such as hyperfibrinolysis or low platelet count, may influence their results. Standardization across platforms is lacking, and their predictive value for clinical outcomes in cirrhosis is not fully established, thus limiting the strength of recommendations in the most recent guidelines [12]. Despite these caveats, VETs provide valuable functional information, aiding the personalized management of coagulation in cirrhotic patients, and can serve as a clinical aid in risk assessment and decision-making.

### 7.4. Is There a Go-to Algorithm for Assessing Bleeding Risk Prior to Interventional Procedures?

**Figure 1:**
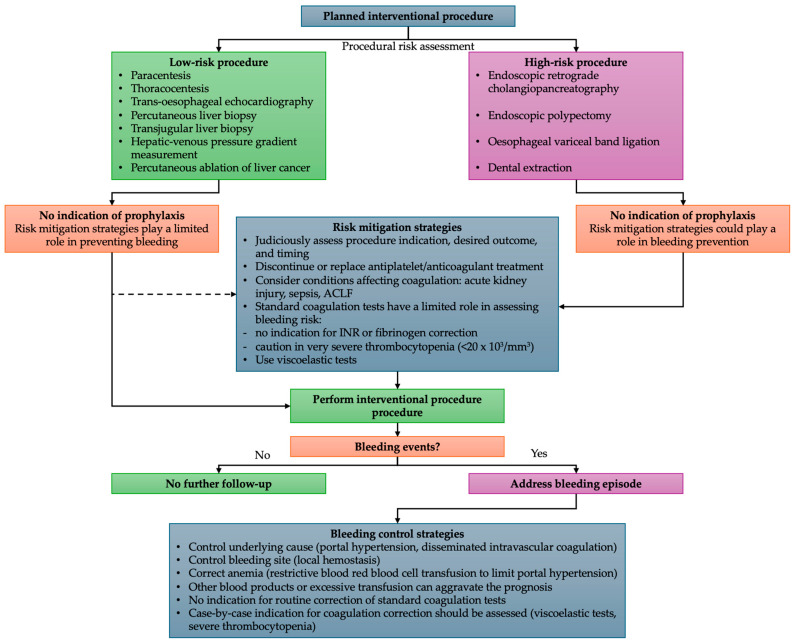
Risk assessment and mitigation algorithm for interventional procedures in patients with cirrhosis.

The short answer to this question is not yet. While there is an ever-increasing evidence pool discussed throughout this article, the strength of the evidence is limited and can only support nuanced recommendations. However, to this point, it is evident that the bleeding risk in patients with cirrhosis is primarily attributed to the type of procedure rather than an intrinsic anticoagulant state, the fact that standard coagulation tests have a limited role in predicting bleeding events, and the fact that the routine prophylactic correction of the standard coagulation profile generates more harm than benefit. In light of the previous discussion, our team proposes the following clinically oriented algorithm to mitigate bleeding risks when performing interventional procedures in patients with advanced liver disease (Figure 1).

## 8. Conclusions

In conclusion, the risk of bleeding in cirrhotic patients undergoing invasive procedures is influenced by a multitude of factors beyond traditional hemostatic parameters such as the INR and platelet count. While thrombocytopenia does not consistently predict bleeding, factors like low fibrinogen levels and AKI have emerged as significant predictors, particularly in patients with ACLF. Current evidence suggests that the routine correction of coagulation abnormalities is often unnecessary, especially for low-risk procedures. However, individualized risk assessments that consider the patient’s clinical context, including the presence of AKI or sepsis, are crucial. A rebalanced approach to hemostasis in cirrhosis underscores the importance of focusing on technical and patient-specific factors rather than relying on conventional coagulation tests alone. Tailored procedural strategies and appropriate clinical management can help mitigate bleeding risks in this vulnerable population.

## Figures and Tables

**Table 1:** Recommendations regarding pre-procedural hemostatic evaluation for liver biopsy.

BSG [28]	- Patients with liver disease undergoing non-lesional biopsies opt for a transvenous route if the international normalized ratio (INR) exceeds 1.4. - For percutaneous lesional biopsies, the INR threshold is below 2.0. - If there is no evidence supporting the efficacy of fresh frozen plasma in mitigating bleeding, its use is not endorsed.
AASLD [29]	- Improve platelet count when under <60.000 per microliter (thrombopoietin receptor agonist/platelet transfusion) or opt for a transvenous route.
Society of Interventional Radiology [30]	- INR threshold < 1.5 to 1.8 for the general population. - INR < 2.5 for those with chronic liver disease.
EFSUMB [31]	- Platelet count < 50.000 prior to liver biopsy—a transfusion is necessary. - INR < 1.5 corrected.

BSG, British Society of Gastroenterology; AASLD, American Association for the Study of Liver Diseases; EFSUMB, European Federation of Societies for Ultrasound in Medicine and Biology; INR, international normalized ratio.

**Table 2:** Pre-procedural bleeding risk assessment in cirrhotic patients.

Standard Coagulation Test	AASLD Recommendation	EASL Recommendation
Platelet Count	- No routine correction.	- No routine correction. - Platelet count 20–50 × 10^9^/L, do not routinely offer platelet transfusion/TPO-R agonist but consider case by case. - Platelet count < 20 × 10^9^/L, consider platelet transfusion/TPO-R agonist case by case.
INR	- No routine correction. - Fresh frozen plasma (FFP) not recommended for patients with prolonged INR. - Use of prothrombin complex concentrate (PCC) discouraged for INR correction.	- No routine correction.
Fibrinogen	- No routine correction.	- Routine correction discouraged.

Abbreviations: FFP—fresh frozen plasma; PCC—prothrombin complex concentrate; TPO-R—thrombopoietin receptor agonist.

**Table 3:** Treatment of active bleeding complications in patients with cirrhosis.

Event	Recommendations
Variceal Bleeding	- Correction of hemostatic abnormalities—not indicated if hemostasis is achieved with portal hypertension-lowering drugs and endoscopic treatment
	- Administration of blood products may increase portal pressure, associated with worse outcomes - A restrictive red blood cell transfusion strategy is beneficial - Large volumes of blood products may increase bleeding
Bleeding due to Portal Hypertension other than Variceal Bleeding	- No studies evaluating correction of hemostasis in cirrhosis patients with active bleeding related to portal hypertension, but not to varices, are available
	- Manage bleeding with portal hypertension-lowering measures; consider correcting hemostasis on a case-by-case basis if bleeding persists despite these measures
Nonportal Hypertensive Cause	- Address active bleeding with local measures and/or interventional radiology procedures
	- Address contributing factors (renal failure, infection/sepsis anemia) - Consider correction of hemostatic abnormalities on a case-by-case basis - Antifibrinolytic agent use is discouraged
